# Optimising graft survival in endothelial keratoplasty for endothelial failure secondary to cytomegalovirus endotheliitis

**DOI:** 10.1186/s12348-019-0180-0

**Published:** 2019-08-02

**Authors:** Milton C. Chew, Donald T. Tan, Soon-Phaik Chee, Lim Li

**Affiliations:** 1 Singapore National Eye Centre, 11 Third Hospital Ave, Singapore, Singapore, 168751 Singapore; 2 Singapore Eye Research Institute, Singapore, Singapore; 3 Department of Ophthalmology, Yong Loo Lin School of Medicine, National University of Singapore, Singapore, Singapore; 4 Duke-NUS Medical School, Singapore, Singapore

**Keywords:** Cytomegalovirus endotheliitis, Endothelial failure, Descemet stripping automated endothelial keratoplasty, Aqueous polymerase chain reaction

## Abstract

**Background:**

There is limited information regarding Descemet stripping automated endothelial keratoplasty (DSAEK) for endothelial failure secondary to cytomegalovirus (CMV) endotheliitis. Treatment is difficult with high recurrence rates. We describe a case when systemic valganciclovir therapy is directed by aqueous CMV-DNA levels, leading to good graft survival.

**Findings:**

A 59-year-old male with bilateral CMV endotheliitis despite antiviral therapy developed endothelial failure and underwent DSAEK. Prior to surgery, aqueous polymerase chain reaction (PCR) for CMV was repeatedly performed, where CMV-positive episodes were treated with systemic valganciclovir. Monthly aqueous analysis was performed until CMV-DNA was undetectable before DSAEK was performed. Post-operative prophylactic systemic valganciclovir treatment was instituted and switched to topical valganciclovir treatment when aqueous samples were negative for CMV.

**Conclusion:**

Targeted aqueous sampling for CMV-DNA perioperatively guides antiviral therapy and ensures adequacy of treatment, minimising the duration of systemic valganciclovir therapy to reduce adverse effects of long-term treatment.

## Introduction

Corneal endotheliitis is a clinical entity characterised by the presence of keratic precipitates (KPs), corneal edema and mild iritis [1]. Cytomegalovirus (CMV) infection has been shown to be an important cause of corneal endotheliitis [2, 3]. If left untreated, it can lead to permanent endothelial damage requiring corneal transplantation. In cases where endothelial keratoplasty has been subsequently performed, recurrence is common despite anti-CMV treatment [4].

In this report, we present a case of bilateral endothelial failure secondary to recurrent CMV endotheliitis that subsequently underwent Descemet stripping automated endothelial keratoplasty (DSAEK). Treatment with anti-CMV therapy during recurrence optimised graft survival.

## Case report

**Fig. 1:**
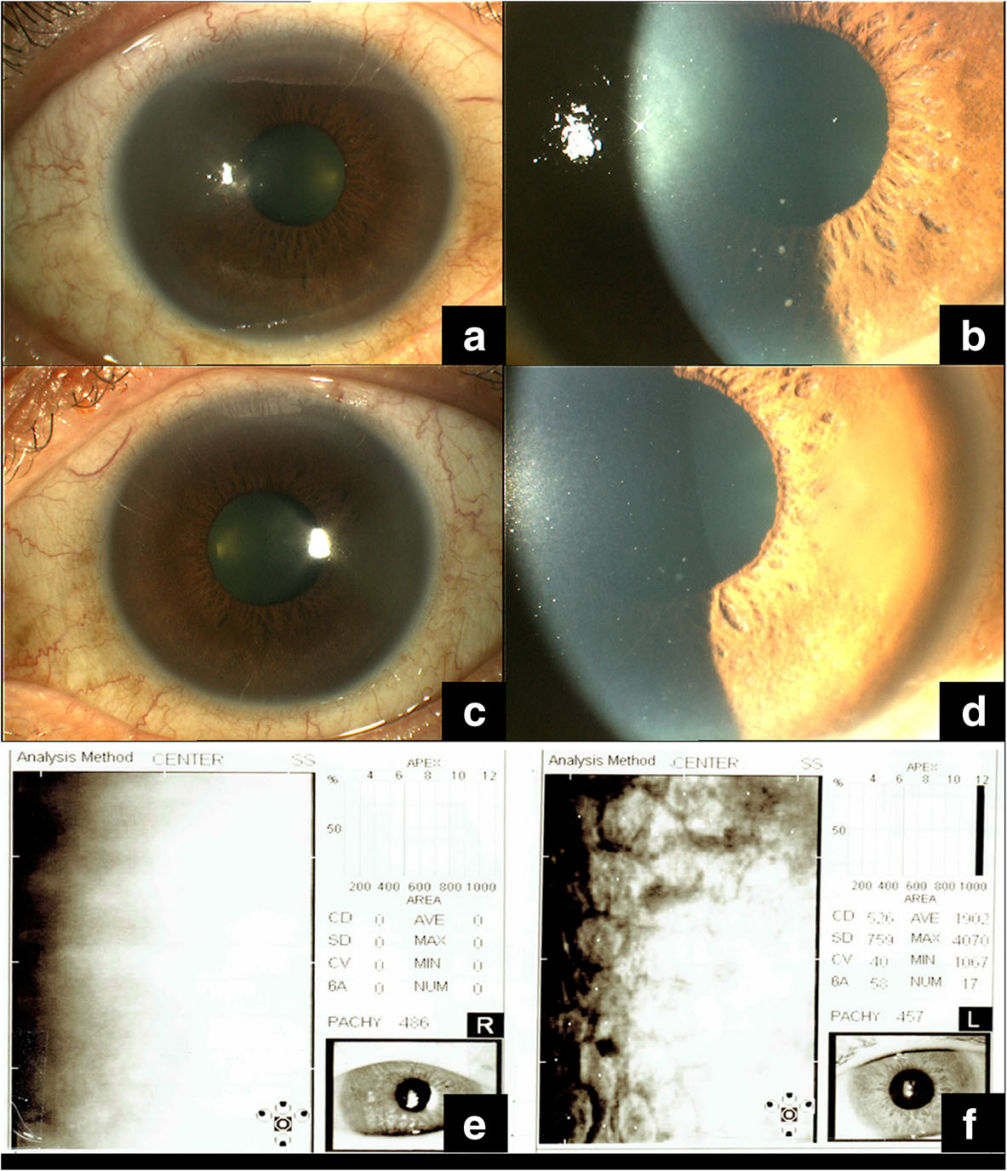
Slit-lamp photographs on initial presentation. Sectorial corneal edema temporally with fine coin-shaped keratic precipitates in the right eye. (**a**, **b**) The left eye with a clear cornea but with similar fine coin-shaped keratic precipitates. (**c**, **d**) The endothelial cell count of both eyes at initial presentation (**e**, **f**)

A 59-year-old gentleman presented in May 2008 for persistent corneal edema, with a history of bilateral recurrent anterior hypertensive uveitis. He had no significant past medical history, nor a history of immunological or infectious diseases of note. He did not have any systemic immunosuppression or significant oncological history. On examination, the best-corrected visual acuity (BCVA) was 6/12 in the right eye (RE) and 6/9 in the left eye (LE). There was right corneal edema with multiple fine coin-shaped keratic precipitates (KPs). His left eye had few KPs (Fig. 1a–d). The right intraocular pressure (IOP) was raised at 35 mmHg, despite being on 3 topical glaucoma medications, and the left was 18 mmHg. Posterior segment examination was unremarkable. Endothelial cell count was unattainable due to corneal edema in his right eye, and 526 cells/mm^2^ in his left eye (Fig. 1e, f).

Aqueous tap was positive for CMV but negative for herpes simplex and varicella zoster virus in both eyes on qualitative polymerase chain reaction (PCR). Quantitative CMV PCR revealed a viral load of 6.3 × 10^4^ copies/ml (RE) and 9.3 × 10^5^ copies/ml (LE). He was co-managed with an infectious disease consultant and treated based on our centre’s treatment regime of oral valganciclovir 900 mg twice daily for 6 weeks, followed by 450 mg twice daily for a further 6 weeks. Topical ganciclovir ophthalmic gel 0.15% (Virgan; Laboratories Théa, Clermount-Ferrand, France) 5 times a day was added. Whilst on systemic therapy, he underwent monthly aqueous taps, which showed decremental CMV-DNA titres. Subsequently, systemic treatment was stopped after 4 months when both eyes became CMV negative.

As the right IOP was poorly controlled despite maximum glaucoma medications, he underwent Ahmed tube implantation on May 2008 and subsequently right trabeculectomy on March 2009. His left eye also developed secondary glaucoma and trabeculectomy was performed in November 2008. Thereafter, his IOPs came under good control.

**Fig. 2:**
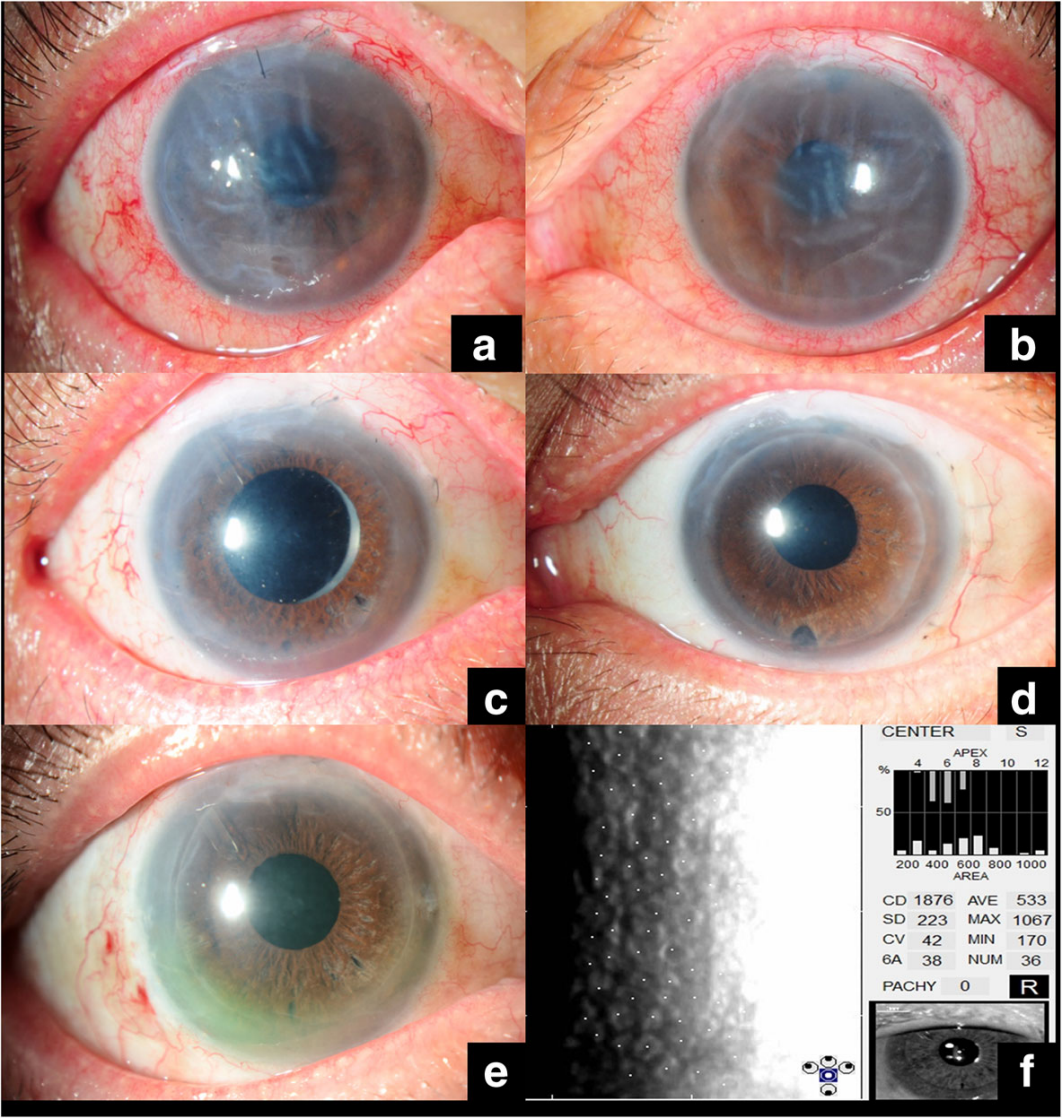
Endothelial failure and corneal decompensation in the right (**a**) and left (**b**) eyes. Clear grafts in the right (**c**) and left (**d**) eyes after successful DSAEK surgery. Clear graft in the right eye (**e**) and latest endothelial cell count (**f**)

By August 2009, he had developed bilateral corneal endothelial failure (Fig. 2a, b). His BCVA dropped to 6/21 in the right eye and 6/15 in the left eye. Pre-operative central corneal thickness was 777 μm in the right eye and 626 μm in the left eye. As the eyes remained quiescent and were CMV negative, and the IOPs were well controlled, DSAEK was offered. He was given CMV prophylaxis with valganciclovir 900 mg twice daily for 3 days pre-operatively, continued for 3 weeks post-operatively whilst the patient was on intensive post-operative topical corticosteroids. The corticosteroid regime consisted of topical prednisolone acetate 1% 3 hourly for 3 weeks, followed by gradual tapering over time. Topical ganciclovir 0.15% ophthalmic gel was also prescribed pre and post-operatively. He successfully underwent right DSAEK on October 2009 and left DSAEK on January 2010. He recovered uneventfully with bilateral clear DSAEK grafts and BCVA of 6/7.5 on the right (Fig. 2c) and 6/6 on the left (Fig. 2d).

At the 2-month post-operatively mark, we performed a routine screening aqueous tap for both eyes. Aqueous CMV-DNA titres demonstrated viral load of 1.4 × 10^4^ copies/ml (RE) and non-detectable (LE) respectively. In view of the recurrence in his right eye, systemic valganciclovir was restarted. Monthly aqueous taps showed decremental CMV titres and were negative for CMV-DNA after completion of 4 months of systemic treatment. He was hence switched to long-term prophylactic topical ganciclovir. He maintained a clear DSAEK graft in both eyes with no recurrence for 3 years with good visual acuity of 6/7.5 in the right and 6/6 in the left.

On January 2014, his right eye developed CMV recurrence. He presented with blurred of vision, with visual acuity of 6/21 in the right eye, localised corneal edema and KPs. IOP was normal. A diagnostic aqueous tap that was performed and showed CMV-DNA viral titres of 110,820 copies/ml. He was restarted on systemic valganciclovir, with a monthly repeated aqueous tap to assess the viral load. After 3 months of treatment by April 2014, the corneal edema had resolved and the graft was clear. Repeat aqueous CMV-DNA was negative. The patient was then switched back to long-term prophylactic topical ganciclovir gel. He continued to have good vision and remained asymptomatic for another year. By May 2015, his right eye vision progressively worsened and eventually developed late graft failure from late endothelial failure, central corneal thickness of 683 μm and normal IOP with a negative aqueous CMV-DNA. He subsequently underwent a repeat right DSAEK on September 2016 with the similar pre-operative and post-operative anti-CMV regime as previously described. On latest review on March 2019, at 2.5 years post-operatively, his BCVA is 6/9 with a clear graft and a good endothelial cell count of 1876cells/mm^2^ (Fig. 2e, f).

His left eye had good vision for 4 years with BCVA of 6/6 after undergoing DSAEK in January 2010. He developed late endothelial failure and corneal decompensation with negative aqueous CMV-DNA by March 2014. He underwent a repeat left DSAEK on September 2014 with the similar pre-operative and post-operative anti-CMV regime as described. However, he did not continue with post-operative systemic valganciclovir due to financial constraints. Subsequently, he developed CMV recurrence with KPs and graft edema, normal IOP and positive aqueous CMV-DNA on January 2016, with subsequent graft failure by September 2016. He had a repeat DSAEK performed on May 2018 with a pre-operative negative aqueous CMV-DNA tap and underwent the similar anti-CMV oral valganciclovir regime. Post-operatively, he recovered uneventfully and IOP was normal. On the latest review on March 2019, his BCVA was 6/9 with a clear graft. As of the latest review, his current medications are topical dexamethasone 0.1% once a day to both eyes.

## Discussion

Cytomegalovirus infection is an important cause of corneal endotheliitis. Seroprevalence ranges from 45–100% [5] and is higher in Asian countries [6]. They usually present unilaterally, but bilateral involvement has been reported [1, 7]. Clinical manifestations include coin-shaped keratic precipitates in a linear pattern, hypertensive anterior uveitis, and progressive endotheliitis (Fig. 1a–d) [2, 3]. This commonly leads to a presumptive misdiagnosis of herpetic keratouveitis [8], with poor response to anti-herpetic treatment. Prompt recognition and treatment with anti-CMV therapy are essential to prevent progressive endothelial deterioration [9].

This case report illustrates that whilst endothelial failure secondary to CMV endotheliitis is an important consideration, endothelial keratoplasty can still be performed with good outcome. We recommend pre-operative aqueous PCR analysis for CMV-DNA prior to planning for corneal transplantation. Patients who are CMV-positive should have systemic valganciclovir initiated. In our centre, the treatment regime for CMV-positive endotheliitis consists of oral valganciclovir 900 mg twice daily for 6 weeks followed by 900 mg once daily for a further 6 weeks [2]. Topical ganciclovir ophthalmic gel is also prescribed 5 times a day [4, 10]. After completing the treatment regime, monthly repeated aqueous taps should be performed to monitor the decremental quantitative CMV-DNA titres. Only when aqueous CMV titres are negative we can stop the systemic treatment and proceed with DSAEK. Post-operatively, our centre recommends 3 weeks of continued prophylactic systemic valganciclovir therapy whilst the patient is on intensive topical corticosteroids to prevent CMV reactivation. As with herpes simplex, which is also associated with reactivation following the stress of surgery, oral antivirals post-operatively help to prevent recurrence [11]. Thereafter, we stop systemic anti-viral therapy too but continue long-term topical ganciclovir therapy to reduce recurrence. We also routinely perform a repeated aqueous sampling at the 2-month post-operative mark to ensure no recurrence.

To date, there is no consensus for the optimal treatment duration for CMV endotheliitis. Systemic valganciclovir treatment has a good response rate but is associated with a high recurrence rate [12]. The recurrence usually occurs after discontinuing of antiviral medications [4]. Furthermore, long-term systemic valganciclovir therapy can lead to serious adverse effects including pancytopenia and myelosuppression [13]. Topical ganciclovir gel has be associated with a lower recurrence rate compared to systemic treatment [12]. However, whilst topical ganciclovir reduces the frequency of recurrence, it was not associated with prolonging the time to recurrence [14]. Therefore, we recommend management to be directed by CMV-DNA aqueous sampling as described above. This way, the duration of systemic ganciclovir can be reduced to minimise side effects, yet with the confidence that the CMV endotheliitis has been adequately treated. Despite anterior chamber paracentesis and sampling being a valuable diagnostic tool, there are concerns regarding rare but potential serious complications of endophthalmitis [15] and trauma to iris and lens. However, it has been shown to be a safe procedure in the hands of an experienced ophthalmologist with adequate aseptic precautions [16, 17].

Whilst we acknowledge the limitations of a case report, we believe that this case highlights an important diagnostic approach in the management of CMV endotheliitis in post-corneal transplant. Corneal endotheliitis is a clinical diagnosis, and a high index of suspicion is warranted when a patient presents with the coin-shaped KPs typically arranged in a linear pattern associated with hypertensive uveitis and localised corneal edema refractory to corticosteroid treatment [2, 3]. However, for patients who have undergone corneal transplantation, it can be difficult to distinguish between CMV reactivation and graft rejection. Inappropriate treatment with corticosteroids may exacerbate the CMV viral infection, increasing inflammation and endothelial damage with subsequent graft failure [10]. Therefore, timely aqueous CMV-DNA analysis can avoid the diagnostic dilemma. In our case, the patient presented with right eye graft edema and keratic precipitates in January 2014. Aqueous sampling confirmed CMV reactivation, and that enabled appropriate systemic antiviral treatment to be initiated early to prevent further endothelial damage and graft failure. The corneal edema resolved with anti-viral treatment and the patient maintained a clear graft with good vision. In contrast, when there was a recurrence of CMV endotheliitis in his left eye in January 2016, his financial constraints and resultant lack of appropriate systemic anti-viral therapy led to irreversible graft failure. The different outcomes between the 2 eyes highlight the importance of instituting early systemic anti-viral therapy in recurrent CMV infection to prevent graft failure.

Late endothelial failure and graft failure eventually occurred in our patient in both eyes despite our best efforts. This highlights the difficulty of management of recurrent CMV endotheliitis post-DSAEK. We advocate firstly the optimisation and control of intraocular pressure from secondary glaucoma prior to transplant, which can adversely affect both the success of subsequent corneal transplant as well as post-operative endothelial health. Early recognition and treatment of rejection or CMV reactivation are essential, with appropriate treatment instituted early. In spite of the above, late endothelial failure still occurred. Pathological alterations of aqueous humour with elevated aqueous cytokines have been postulated to contribute to endothelial cell loss after DSAEK [18]. The exact mechanism of corneal endotheliitis is still unknown, but autoimmune processes play an important role in the pathophysiology of CMV endotheliitis, with anterior chamber-associated immune deviation stimulated during intermittent reactivation of the CMV virus [19].

Endothelial keratoplasty has largely overtaken penetrating keratoplasty (PK) as the treatment of choice for endothelial disease, including CMV endotheliitis due to its superior safety profile, as well as more rapid and predictable visual outcomes as compared to PK. Many of the disadvantages of PK, such as suture-related complications, graft rejection and wound dehiscence, are also greatly reduced in DSAEK [20].

In summary, we illustrate the management of a patient who underwent endothelial keratoplasty for endothelial failure secondary to CMV endotheliitis. Targeted aqueous sampling for CMV-DNA titres preoperatively and help optimise graft timing and postoperatively to guide treatment. This ensures the adequacy of treatment and minimises the duration of systemic valganciclovir therapy to reduce the adverse effects of long-term treatment.

## Data Availability

Please contact the corresponding author for data requests.

## Abbreviations

BCVA: Best-corrected visual acuity
CMV: Cytomegalovirus
DSAEK: Descemet stripping automated endothelial keratoplasty
IOP: Intraocular pressure
KPs: Keratic precipitates
PCR: Polymerase chain reaction

## References

[1] Khodadoust AA, Attarzadeh A (1982). Presumed autoimmune corneal endotheliopathy. Am J Ophthalmol.

[2] Chee S-P, Bacsal K, Jap A (2007). Corneal endotheliitis associated with evidence of cytomegalovirus infection. Ophthalmology.

[3] Koizumi N, Suzuki T, Uno T (2008). Cytomegalovirus as an etiologic factor in corneal endotheliitis. Ophthalmology.

[4] Ang M, Sng CCA, Chee S-P (2013). Outcomes of corneal transplantation for irreversible corneal decompensation secondary to corneal endotheliitis in Asian eyes. Am J Ophthalmol.

[5] Cannon MJ, Schmid DS, Hyde TB (2010). Review of cytomegalovirus seroprevalence and demographic characteristics associated with infection. Rev Med Virol.

[6] Wong A, Tan KH, Tee CS, Yeo GS (2000). Seroprevalence of cytomegalovirus, toxoplasma and parvovirus in pregnancy. Singapore Med J.

[7] Koizumi N, Inatomi T, Suzuki T (2015). Clinical features and management of cytomegalovirus corneal endotheliitis: analysis of 106 cases from the Japan corneal endotheliitis study. British Journal of Ophthalmology.

[8] van Boxtel LAA, van der Lelij A, van der Meer J, Los LI (2007). Cytomegalovirus as a cause of anterior uveitis in immunocompetent patients. Ophthalmology.

[9] Choi JA, Kim KS, Jung Y et al (2016) Cytomegalovirus as a cause of hypertensive anterior uveitis in immunocompetent patients. J Ophthalmic Inflamm Infect 6. 10.1186/s12348-016-0100-5 10.1186/s12348-016-0100-5 PMC5017963 27613273

[10] Anshu A, Chee S-P, Mehta JS, Tan DTH (2009). Cytomegalovirus endotheliitis in Descemet’s stripping endothelial keratoplasty. Ophthalmology.

[11] Agrawal Rupesh, Murthy Somashiela, Ganesh Sudha K., Phaik Chee Soon, Sangwan Virender, Biswas Jyotimai (2012). Cataract Surgery in Uveitis. International Journal of Inflammation.

[12] Chee S-P, Jap A (2010). Cytomegalovirus anterior uveitis: outcome of treatment. Br J Ophthalmol.

[13] Fellay J, Venetz J-P, Aubert J-D (2005). Treatment of cytomegalovirus infection or disease in solid organ transplant recipients with valganciclovir. Transplant Proc.

[14] Wong JXH, Agrawal R, Wong EPY, Teoh SC (2016) Efficacy and safety of topical ganciclovir in the management of cytomegalovirus (CMV)-related anterior uveitis. J Ophthalmic Inflamm Infect 6. 10.1186/s12348-016-0078-z 10.1186/s12348-016-0078-z PMC4791412 26976016

[15] Helbig H, Noske W, Kleineidam M (1995). Bacterial endophthalmitis after anterior chamber paracentesis. Br J Ophthalmol.

[16] Trivedi D, Denniston AKO, Murray PI (2011). Safety profile of anterior chamber paracentesis performed at the slit lamp. Clin Experiment Ophthalmol.

[17] Van der Lelij A, Rothova A (1997). Diagnostic anterior chamber paracentesis in uveitis: a safe procedure. Br J Ophthalmol.

[18] Yazu H, Yamaguchi T, Aketa N (2018). Preoperative aqueous cytokine levels are associated with endothelial cell loss after Descemet’s stripping automated endothelial keratoplasty. Invest Ophthalmol Vis Sci.

[19] Suzuki T, Ohashi Y (2008). Corneal endotheliitis. Semin Ophthalmol.

[20] Tan DTH, Dart JKG, Holland EJ, Kinoshita S (2012). Corneal transplantation. Lancet.

